# From the wound to the bench: exoproteome interplay between wound-colonizing *Staphylococcus aureus* strains and co-existing bacteria

**DOI:** 10.1080/21505594.2017.1395129

**Published:** 2018-03-01

**Authors:** Andrea N. García-Pérez, Anne de Jong, Sabryna Junker, Dörte Becher, Monika A. Chlebowicz, José C. Duipmans, Marcel F. Jonkman, Jan Maarten van Dijl

**Affiliations:** a Department of Medical Microbiology, University of Groningen, University Medical Center Groningen, Hanzeplein 1, Groningen, the Netherlands; b Department of Molecular Genetics, Groningen Biomolecular Sciences and Biotechnology Institute, University of Groningen, AG Groningen, the Netherlands; c Institute for Microbiology, Ernst-Moritz-Arndt Universität Greifswald, Friedrich-Ludwig-Jahn-Str. 15, Greifswald, Germany; d Department of Dermatology, University of Groningen, University Medical Center Groningen, Hanzeplein 1, RB Groningen, the Netherlands

**Keywords:** *Staphylococcus aureus*, *Bacillus thuringiensis*, *Klebsiella oxytoca*, co-culture, exoproteome, chronic wound

## Abstract

Wound-colonizing microorganisms can form complex and dynamic polymicrobial communities where pathogens and commensals may co-exist, cooperate or compete with each other. The present study was aimed at identifying possible interactions between different bacteria isolated from the same chronic wound of a patient with the genetic blistering disease epidermolysis bullosa (EB). Specifically, this involved two different isolates of the human pathogen *Staphylococcus aureus*, and isolates of *Bacillus thuringiensis* and *Klebsiella oxytoca*. Particular focus was attributed to interactions of *S. aureus* with the two other species, because of the high staphylococcal prevalence among chronic wounds. Intriguingly, upon co-cultivation, none of the wound isolates inhibited each other's growth. Since the extracellular proteome of bacterial pathogens is a reservoir of virulence factors, the exoproteomes of the staphylococcal isolates in monoculture and co-culture with *B. thuringiensis* and *K. oxytoca* were characterized by Mass Spectrometry to explore the inherent relationships between these co-exisiting bacteria. This revealed a massive reduction in the number of staphylococcal exoproteins upon co-culturing with *K. oxytoca* or *B. thuringiensis*. Interestingly, this decrease was particularly evident for extracellular proteins with a predicted cytoplasmic localization, which were recently implicated in staphylococcal virulence and epidemiology. Furthermore, our exoproteome analysis uncovered potential cooperativity between the two different *S. aureus* isolates. Altogether, the observed exoproteome variations upon co-culturing are indicative of unprecedented adaptive mechanisms that set limits to the production of secreted staphylococcal virulence factors.

## Introduction

Studies on pathogenic bacteria, either *in vitro* or in animal models, commonly address the respective bacteria as secluded species. Such studies do not consider the natural ecosystem of bacteria that colonize and invade the human body, which may happen in competition or cooperation with the existing microbiota and other pathogens. Furthermore, although bacteria-host interactions are the key to understanding the pathophysiology of infectious diseases, it is also important to scrutinize the parallel relationships between different bacterial species. This segment of microbial ecology may also elucidate how pathogens adjust their metabolism and other adaptive responses to survive and persist in the human body.

An example of a well-adapted bacterium associated with humans and animals, both as a commensal and as a pathogen, is *Staphylococcus aureus* [1, 2]. This bacterium has an exceptional ability to adapt and evolve swiftly into multidrug resistant lineages, as shown since the clinical introduction of penicillin and subsequently developed antibiotics [3, 4]. *S. aureus* is able to cause a wide range of afflictions ranging from impetigo and upper respiratory tract infections to osteomyelitis, endocarditis and sepsis. The fruitless attempts to develop a vaccine against *S. aureus *[5, 6]. suggest the need for a better understanding of community interactions and the respective metabolic networks. This need is further underpinned by the high prevalence of infections caused by methicillin-resistant *S. aureus* (MRSA) in hospitals and communities worldwide [7, 8]. Nevertheless, the vast majority of molecular studies on the virulence of *S. aureus* have investigated this pathogen in isolation.

In the present study, we explored the possible interactions between two phenotypically different *S. aureus* isolates from a chronic wound of a patient with epidermolysis bullosa (EB) and two other bacterial species, *Bacillus thuringiensis* and *Klebsiella oxytoca*, isolated from the same wound. EB is a genetic blistering disease characterized by the development of chronic wounds upon simple mechanical trauma. It was previously shown that *S. aureus* is an important wound colonizer in patients with EB, where a patient can carry several *S. aureus* types in a single wound, and autoinoculation from the upper respiratory tract can occur [9–15]. Notably, next to *S. aureus*, also other bacteria are known to colonize the wounds of EB patients [16]. Considering these colonization characteristics and the high prevalence of *S. aureus* in the chronic wounds of patients with EB [15], we decided to study the interactions of *S. aureus* wound isolates with coexisting *B. thuringiensis* or *K. oxytoca* isolates. In particular, we focused attention on the extracellular proteomes (in short, exoproteomes) of these bacteria, because the exoproteome is a major reservoir of virulence factors [17].

*B. thuringiensis* is a Gram-positive, spore-forming aerobic bacterium that is commonly used as a safe bioinsecticide since it produces pore-forming proteins that are toxic for insect larvae [18]. It belongs to the same group as *Bacillus anthracis* and *Bacillus cereus*, the etiologic agents of anthrax and the “fried rice syndrome” (i.e. food poisoning), respectively [19, 20]. *B. thuringiensis*, unlike the other two, is not known as a major human pathogen [21]. However, it has been reported that this bacterium may be responsible for opportunistic pulmonary infections in susceptible subjects [22, 23]. On the other hand, the Gram-negative bacterium *K. oxytoca* is clearly regarded as an opportunistic pathogen. It is commonly found on mucosal surfaces of mammals, including the nasopharynx and colon of humans [24]. *Klebsiella* species are the second most common cause of nosocomial Gram-negative bacteremia after *Escherichia coli*, and they are often involved in urinary tract infections in risk group patients and neonatal sepsis [25–28].

Altogether, the present study was aimed at exploring the potentially synergistic or competitive relationships between *S. aureus* and two different classes of bacteria that are, in principle, capable of colonizing and invading the human body, thereby causing disease. Our specific objective was to unravel the bacterial response patterns using the exoproteome as a read-out. To this purpose, two different *S. aureus* isolates were, respectively, co-cultured with *B. thuringiensis* (Bt) or *K. oxytoca* (Ko) isolates from the same wound environment, and the exoproteomes of individually or co-cultured bacteria were analyzed by Mass Spectrometry (MS).

## Results

### S. *aureus* grows unimpaired when co-cultured with *B. thuringiensis* or *K. oxytoca* on solid agar

**Figure 1.**
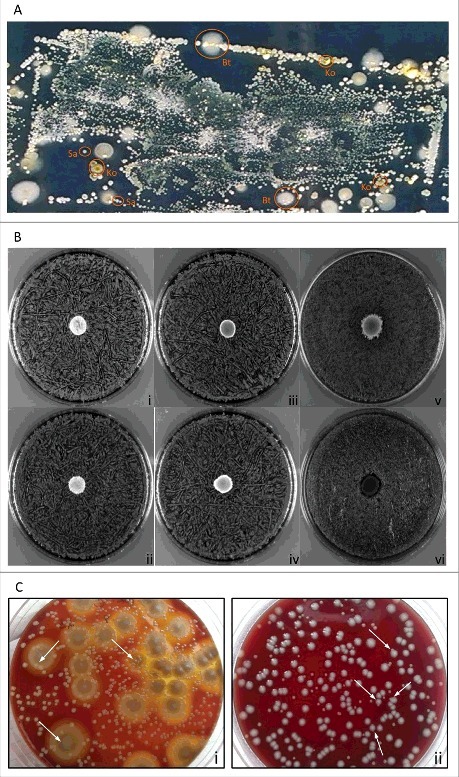
Growth characteristics of the isolated bacteria on agar plates. (A) Microbiome topography of culturable bacteria from the wound dressing of a patient with Junctional EB. The used dressing from a chronic wound was replica plated onto CLED agar for isolation of the bacteria present. (B) Zone inhibition experiments. Unimpaired staphylococcal growth upon spotting of *B. thuringiensis* or *K. oxytoca*: (i) t111+Bt, (ii) t13595+Bt, (iii) t111+Ko, and (iv) t13595+Ko. Unimpaired growth of *K. oxytoca* upon spotting of *B. thruingiensis* (v). *S. aureus* growth inhibition halo caused by spotting of *B. subtilis* 168 onto a lawn of staphylococcal cells (vi; both *S. aureus* strains exhibited the same effect). (C) Dilution and subsequent plating: (i) *S. aureus* colonies growing on top of larger colonies of *B. thuringiensis*; (ii) *K. oxytoca* and *S. aureus* colonies growing in close proximity and occasionally touching each other. Typical *S. aureus* colonies are marked with arrows.

In a previous study, we applied replica plating of used wound dressings to assess the topography of distinct *S. aureus* types in chronic wounds of patients with EB [13]. Notably, this previous study was focused on *S. aureus* only, and it did not take into consideration other co-existing microbial species that are clearly evident upon replica plating of wound dressings (Fig. 1A). Such co-existing species were collected in the present study, using the dressing of a chronic wound from a patient with Junctional Epidermolysis Bullosa. This led to the identification of two *S. aureus* isolates with *spa* types t111 and t13595 that grew in close proximity to *B. thuringiensis* (Bt) and *K. oxytoca* (Ko) isolated from the same wound. A zone inhibition experiment was employed to assess possible competitive or bactericidal interactions between these four wound isolates. As shown for the *S. aureus* t111 and t13595 isolates in Fig. 1B, there was no growth inhibition detectable when, respectively, Bt or Ko were spotted onto blood agar plates that were confluently inoculated with *S. aureus*. The same was essentially observed when Bt was spotted onto a confluent inoculated plate with Ko. Additionally, *B. subtilis* strain 168 was chosen as control based on the known growth inhibition that *B. subtilis* 168 exerts on *S. aureus* due to production of the bacteriocin sublancin [29]. Indeed, the spotting of *B. subtilis* 168 onto the *S. aureus* cells caused a clear growth inhibition zone (Fig. 1B). Furthermore, it was noted that the growth of Bt and Ko occurred on top of the *S. aureus* spread. To confirm this observation, serial dilution experiments were performed with mixed cultures of *S. aureus* and Bt, or *S. aureus* and Ko. As marked by the arrows in Fig. 1, colonies of *S. aureus* were not only able to grow in the proximity of Bt and Ko colonies, but some were observed to touch or even grow on top of each other (Fig. 1C). This is fully consistent with the situation encountered in the wound environment from which the bacteria were isolated and where they thrive in close proximity (Fig. 1A).

### S. *aureus* isolates t111 and t13595 are highly related but not identical

As a first step in the characterization of the selected *S. aureus* t111 and t13595 isolates, their antibiotic resistance profiles were determined. Both isolates were shown to be resistant to tetracycline, ciprofloxacin and fusidic acid. In addition, isolate t111 was found to be resistant to trimethoprim and sulfamethoxazole (Supplemental Table S1). These findings suggested that both isolates are very similar, but not identical. This was confirmed by whole-genome sequencing, revealing that the respective core genomes differed only by 192 nucleotides. Genome sequencing further revealed that both *S. aureus* isolates belong to the sequence type 5 (ST5). Thus, the closest related reference genome is the one of *S. aureus* strain N315 which was, therefore, used for comparative genomic analyses and annotation.

Although there are not many differences in the core genomes of the t111 and t13595 isolates, a few of these differences are noteworthy. For example, the *fnbA* and *fnbB* genes of the t111 isolate were recombined into a single gene. Isolate t13595 contained a point mutation in *agrA* specifying the replacement of Gly148 with Asp, and this mutation was correlated with an *agr* negative phenotype (i.e. unlike the t111 isolate, the t13595 isolate displayed no α, β or δ-hemolysis). Frame shift mutations were also observed in various other genes of the t13595 isolate, including the *mutL* gene for a DNA mismatch repair protein, and genes such as *sbi, araC, geh* and *fnbA*. For a more detailed comparison of the *S. aureus* isolates with respect to different mobile genetic elements, please refer to Supplemental Table S2.

### Total numbers of *S. aureus* exoproteins are reduced when *S. aureus* is co-cultured with Bt or Ko

Since the *S. aureus*, Bt and Ko isolates addressed in this study were co-existent in the same wound environment and are capable of growing in close contact in an *in vitro* setting, we wanted to know whether and, if so, how they influence each other. To address this question, we selected a proteomics approach in which we focused our attention on the extracellular proteome. Further, to simulate the conditions in the human body, we selected the tissue culture medium RPMI as the growth medium for our studies, because *S. aureus* cells grown on RPMI or human plasma display very similar genome-wide transcript profiles [30]. Thus, the *S. aureus* isolates t111 and t13595 were cultured in three different conditions, namely in pure culture, or in combination with either Bt or Ko. As expected from the plating experiments, we observed only slight differences in the logarithmic growth rates of the mixed cultures as assessed by OD_600_ readings. For example, when *S. aureus* was co-cultured with Bt, the overall growth seemed slightly slower, while the opposite happened when *S. aureus* was co-cultured with Ko (Figure S1). This can be explained by the particular growth rates of the Bt and Ko isolates on RPMI, where the growth rate of Bt in monoculture is relatively slow, while that of Ko is relatively fast. Nevertheless, the overall growth patterns of the co-cultures were similar to those of the *S. aureus* pure cultures and, importantly, they were highly reproducible. Hence, in all the experiments, our reference for sampling was the *S. aureus* growth curve in monoculture.

**Figure 2.**
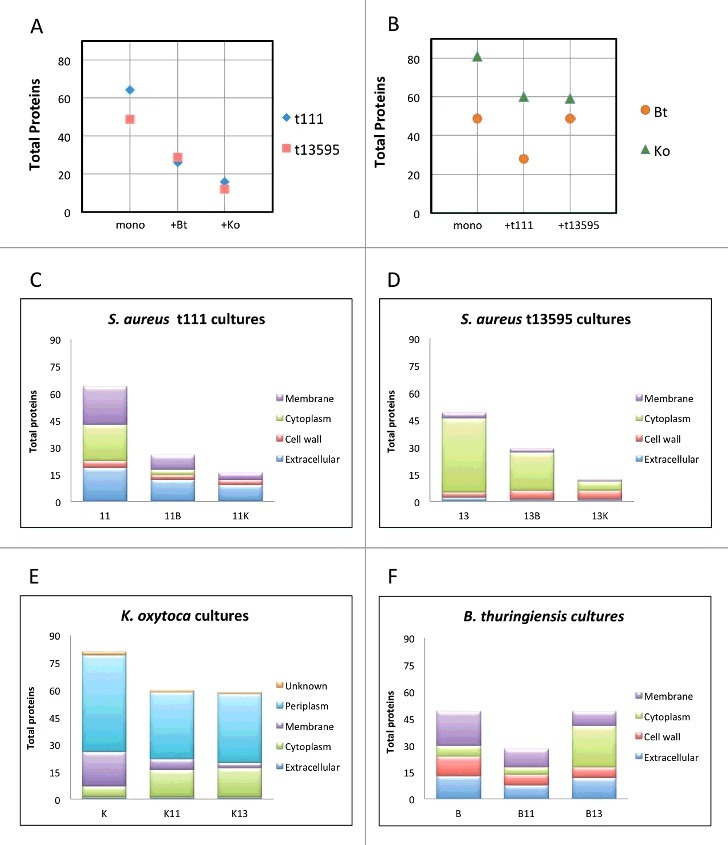
Total numbers of extracellular proteins identified per culture. (A) Numbers of *S. aureus* t111 and t13595 exoproteins identified in monocultures or upon co-culturing with *B. thuringiensis* (+Bt) or *K. oxytoca* (+Ko). (B) Numbers of *B. thuringiensis* and *K. oxytoca* exoproteins identified in monocultures or upon co-culturing with *S. aureus* t111 (+t111) or t13595 (+t13595) isolates. (C-F) Predicted subcellular localization of proteins per strain in mono- or co-cultures. (C) 11, *S. aureus* t111 exoproteins; 11B, *S. aureus* t111 exoproteins upon co-culture with *B. thuringiensis*; 11K, *S. aureus* t111 exoproteins upon co-culture with *K. oxytoca*. (D) 13, *S. aureus* t13595 exoproteins; 13B, *S. aureus* t13595 exoproteins upon co-cultures with *B. thuringiensis*; 13K, *S. aureus* t13595 exoproteins upon co-culture with *K. oxytoca*. (E) K, *K. oxytoca* exoproteins; K11, *K. oxytoca* exoproteins upon co-culture with *S. aureus* t111; K13, *K. oxytoca* exoproteins upon co-culture with *S. aureus* t13595. (F) B, *B. thuringiensis* exoproteins; B11, *B. thuringiensis* exoproteins upon co-culture with *S. aureus* t111; B13, *B. thuringiensis* exoproteins upon co-culture with *S. aureus* t13595.

To obtain an overview of the behavior of the cultures, we compared the total numbers of proteins detected by LC-MS/MS in all conditions (Fig. 2A,B) and verified the reproducibility of the biological replicates (Supplemental Figure S2). This analysis revealed clear differences between the pure and mixed cultures. Particularly, a total of 64 extracellular proteins were detected in the *S. aureus* t111 monoculture, while in combination with Bt only 26 extracellular staphylococcal proteins were detected, and merely 16 when the t111 isolate was cultured with Ko. Essentially the same was observed for the exoproteome of the strain t13595, where 49 extracellular proteins were found in the monoculture, and only 29 and 12 in the co-cultures with Bt or Ko, respectively (Fig. 2A). We also assessed the numbers of extracellular proteins detected in the monocultures of Bt and Ko in comparison to the co-cultures. The results indicate a decrease of ∼25% in the number of Ko proteins found in the co-cultures with *S. aureus* (Fig. 2B), but this effect was clearly not as prominent as the effect of Ko on the staphylococcal exoproteome. On the other hand, the number of exoproteins of Bt decreased only in the presence of the *S. aureus* t111 isolate but not in the presence of the t1359 isolate (Fig. 2B). Of note, the exoprotein differences among the cultures were also reflected in the absolute amounts of protein detectable in the growth medium (Figure S3).

As a first approach to characterize the identified extracellular proteins, we assessed their predicted subcellular localization. For the two *S. aureus* isolates and the Bt isolate, which are all three Gram-positive bacteria, altogether 150 (50%) predicted cytoplasmic proteins, 26 (9%) cell wall proteins, 74 (25%) membrane proteins and 47 (16%) extracellular proteins were identified in the exoproteome (Supplemental Figure S4). For the Gram-negative Ko isolate, we detected 50 (34%) predicted cytoplasmic, 25 (17%) membrane, 1 (1%) extracellular, and 67 (46%) periplasmic proteins (Supplemental Figure S4). Because of the acute changes in the *S. aureus* exoproteome upon co-culturing, we were interested in knowing which predicted subcellular protein fractions were most strongly affected in the various conditions. For both *S. aureus* isolates, the fraction of predicted cytoplasmic proteins sharply decreased upon co-culturing, and such proteins were even completely missing from the co-culture of the t111 isolate with Ko (Fig. 2C,D). On the contrary, the fraction of predicted cytosolic Bt proteins in the extracellular proteome sharply increased when co-cultured with *S. aureus* t13595, while the numbers of predicted cytoplasmic proteins of Ko in co-cultures with either of the two *S. aureus* isolates were similar, showing a relatively slender increase when *S. aureus* was present (Fig. 2E,F).

### Co-cultured *S. aureus, B. thuringiensis* and *K. oxytoca* isolates display species-specific exoproteome changes in terms of biological processes

**Figure 3.**
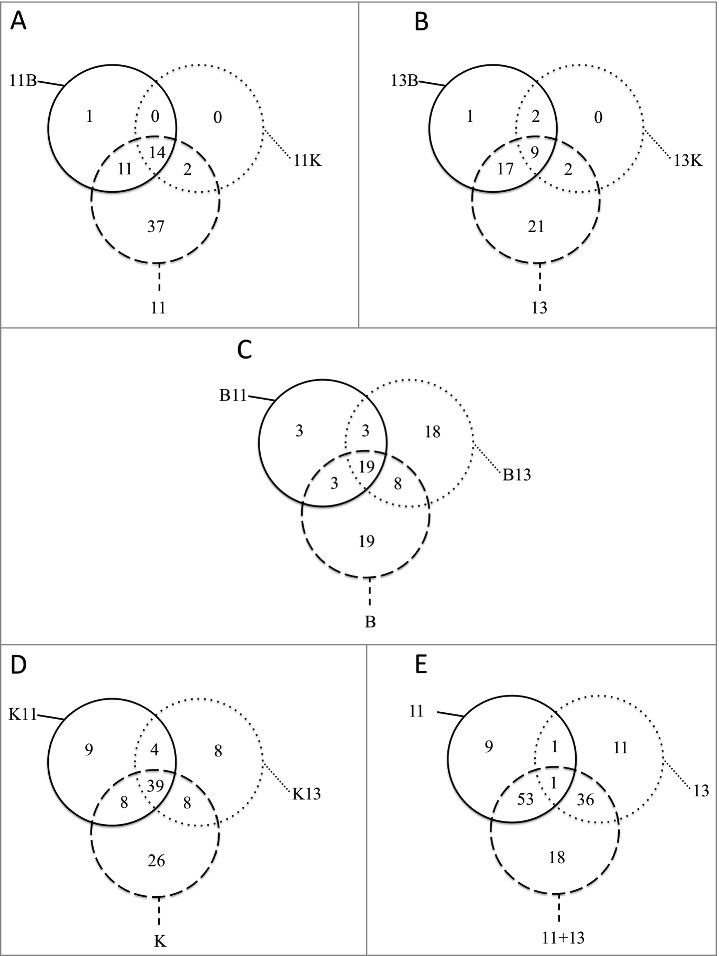
Relationships among cultures. The diagrams show the number of proteins identified in all cultures and those proteins shared in the different conditions. (A) Total number of *S. aureus* t111 proteins in monoculture (11) and in co-culture with *B. thuringiensis* (11B) or *K. oxytoca* (11K); (B) Total number of *S. aureus* t13595 proteins in monoculture (13) and in co-culture with *B. thuringiensis* (13B) or *K. oxytoca* (13K); (C) Total number of *B. thuringiensis* proteins in monoculture (B) and in co-culture with *S. aureus* t111 (B11) or *S. aureus* t13595 (B13); (D) Total number of *K. oxytoca* proteins in monoculture (K) and in co-culture with *S. aureus* t111 (K11) or *S. aureus* t13595 (K13); (E) Total number of *S. aureus* proteins in monoculture (11) or (13) and in co-culture (11+13).

Although both *S. aureus* isolates are closely related, particular variations in their exoproteomes were observed. For example, the core exoproteome of the t111 isolate included only 14 proteins (i.e. proteins constant in all cultures), while the core exoproteome of the t13595 isolate included only nine proteins (Fig. 3 and Table S3). Likewise, the biological processes in which these proteins are involved were also different. The core exoproteome of the t111 isolate was characterized by functions in peptidoglycan catabolic processes, pathogenesis and cobalamin transport, whereas the core exoproteome of the t13595 isolate is involved in cell redox homeostasis, cell wall organization, glucose metabolic processes, pathogenesis, cell adhesion and glycine betaine biosynthetic processes from choline.

Remarkably, there were not many staphylococcal proteins expressed uniquely in the co-cultures. In fact, there were no distinctive proteins detected in the co-cultures with Ko for both *S. aureus* isolates (Fig. 3A,B). For the co-cultures with Bt, we identified only the translocase subunit SecG as a distinctive feature in the growth medium of the t111 isolate, and an uncharacterized distinctive protein was identified in the growth medium of the t13595 isolate. In contrast, in the presence of the t111 or t13595 isolates, Ko displayed 9 and 8 unique extracellular proteins, respectively (Fig. 3D). The latter 9 proteins produced in the presence of the t111 isolate were related, by their gene ontologies, to chromosome condensation, regulation of transcription, the phosphoenolpyruvate-dependent sugar phosphotransferase system, and glycerol ether metabolic processes. Similarly, Bt displayed 18 distinctive extracellular proteins in the presence of the t13595 isolate (Fig. 3C). These proteins relate to iron-sulphur cluster assembly, protein transport, cell cycle, glucose and glycerol ether metabolic processes.

To further categorize the identified extracellular proteins, we classified them according to their function and categorized them in one of the following six groups: (1) cell envelope and cellular processes (including cell wall/membrane, transport/binding proteins and lipoproteins, signal transduction, membrane bioenergetics, motility and chemotaxis, protein secretion, cell division, cell adhesion, response to stress); (2) information pathways (including DNA restriction/modification and repair, transcription and regulation, ribosomal proteins, protein synthesis/modification/folding); (3) intermediary metabolism; (4) cell redox homeostasis, (5) virulence factors; and (6) unknown functions (see Supplemental Table S3).

**Figure 4.**
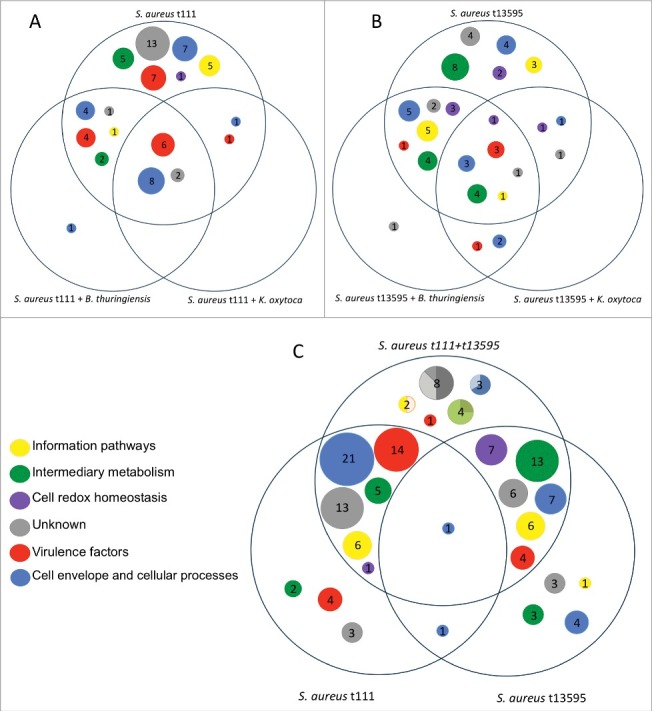
Overview of the predicted functions of the exoproteins found in all staphylococcal cultures. Diagram (A) depicts only *S. aureus* t111 proteins. The area on the top shows the proteins detected only in monoculture, while the lower left area shows a single protein detected when t111 was co-cultured with *B. thuringiensis*. Likewise, diagram (B) shows exclusively the proteins that belong to *S. aureus* t13595 in monoculture and co-culture with *B. thuringiensis* or *K. oxytoca*. Diagram (C) depicts the proteins of *S. aureus* t111 and t13595 grown in monoculture in the bottom areas, whilst the upper area shows the proteins identified upon co-culturing both *S. aureus* isolates. In the latter area, the circles are represented as pie charts. The charts show the percentage of proteins that belong to each isolate. For the unknown proteins (in grey), 50% belong to t111, 37% to t13595, and 12.5% to both of them; for the cell envelope proteins (blue) 67% belong to t111 and 33% to both isolates; for proteins involved in intermediary metabolism (green), 25% belong to t111 and 75% to t13595; and for information pathways proteins (yellow), 50% belong to t111 and 50% to both isolates.

In the diagrams of Fig. 4, the differences between the *S. aureus* t111 and t13595 isolates in terms of the functions of extracellular proteins are clearly evident; while the t111 isolate seems to be more virulent, the extracellular proteins of the t13595 are more involved in processes related to cell redox homeostasis and intermediary metabolism. Further, the core functions of the t13595 isolate appear more diverse than those of the t111 isolate. In the case of Ko, the overall picture indicates that this bacterium's exoproteome is strongly involved in cell envelope and cellular processes, especially processes relating to the transport and binding of proteins and lipoproteins (Supplemental Figure S5A). For Bt, we observed that the response towards the co-cultured *S. aureus* isolates was considerably different. In particular, the extracellular Bt proteins related to many more different functions when Bt was co-cultured with the t13595 isolate than when Bt was co-cultured with the t111 isolate. Interestingly, like the *S. aureus* t13595 isolate co-cultured with Bt, Bt also exhibited many extracellular proteins related to redox homeostasis when co-cultured with the t13595 isolate (Supplementary Figure S5B).

### Particular exoproteins of *S. aureus, K. oxytoca* and *B. thuringiensis* show quantitative changes in mono- or co-cultures

Conceivably not all the differences in the exoproteome composition of mono- and co-cultured *S. aureus*, Ko and Bt are absolute, but also quantitative differences may exist for particular exoproteins. Therefore, we comparatively assessed significant fold-changes in the exoproteins detected in mono- and co-cultures by mapping their spectral counts from all replicates using volcano plots (Supplemental Figure S6). The results of this analysis, presented in Table 1, indicate that there were 11 exoproteins significantly ‘upregulated’ in the *S. aureus* monocultures compared to the co-cultures with Bt or Ko. These included *S. aureus* proteins, such as elongation factor Ts, triosephosphate isomerase, thioredoxin reductase, 2,3-bisphosphoglycerate-dependent phosphoglycerate mutase and a secreted β-lactamase. In the case of Ko, 22 exoproteins were present in significantly different amounts when mono and co-cultures were compared. Here, 17 exoproteins of Ko were significantly upregulated in the monocultures, whereas 5 other exoproteins of Ko were significantly upregulated in the co-cultures with *S. aureus*. The latter proteins are mostly involved in carbohydrate metabolism. Also in the case of Bt, 22 exoproteins were present in significantly different amounts when mono and co-cultures were compared (Table 1). Interestingly, 21 of these Bt exoproteins were significantly ‘upregulated’ in the monocultures compared to the co-cultures with *S. aureus*. The functions of these proteins were rather diverse, including the cell wall hydrolase LytE, three different proteases, and the protein folding catalyst PrsA.

**Table 1.** Summary of differentially detected extracellular proteins among the different cultures. Volcano plots (Supplemental Figure S6) displayed *P* values (-log_10_) versus fold changes (log_2_) of the normalized spectral counts. Folds ≤1 represent proteins that were significantly up-regulated in the co-cultures and folds >1 represent proteins that were significantly up-regulated in monoculture.

Species	Protein	Function / Gene ontology	Fold	p value
*S. aureus*	Beta-lactamase	Hydrolysis of beta-lactamic ring	12.81	0.041
*S. aureus*	Phosphocarrier protein HPr	Phosphoenolpyruvate-dependent sugar phosphotransferase system. Serine/threonine kinase activity. Regulation of transcription, DNA-templated	6.61	0.009
*S. aureus*	Fructose-bisphosphate aldolase class 1	Glycolytic process	12.89	0.042
*S. aureus*	Elongation factor Ts	Binds to aminoacyl-tRNA. Protein biosynthesis	29	0.034
*S. aureus*	Triosephosphate isomerase	Gluconeogenesis. Glycolytic process. Pentose-phosphate shunt	18	0.038
*S. aureus*	2,3-bisphosphoglycerate-dependent phosphoglycerate mutase	Gluconeogenesis. Glycolytic process	16.80	0.048
*S. aureus*	Cysteine synthase	Cysteine biosynthetic process from serine	13	0.045
*S. aureus*	Phosphate acetyltransferase	Acetyl-CoA biosynthetic process	15.60	0.039
*S. aureus*	Thioredoxin reductase	Removal of superoxide radicals	17	0.040
*S. aureus*	Putative universal stress protein SSP1056	Response to stress	13	0.046
*S. aureus*	D-alanine-poly(phosphoribitol) ligase subunit 2	Cell wall organization. Lipoteichoic acid biosynthetic proces. Regulation of cell shape	6	0.039
*K. oxytoca*	Uncharacterized protein	Unknown	2.68	0.003
*K. oxytoca*	Colicin I receptor	Bacteriocin transport. Iron assimilaiton. Siderophore transmembrane activity	1.63	0.036
*K. oxytoca*	Glyceraldehyde-3-phosphate dehydrogenase	Glucose metabolic process. Glycolytic process	0.33	0.044
*K. oxytoca*	TonB-dependent hemin, ferrichrome receptor	Transporter activity	1.79	0.024
*K. oxytoca*	Glutathione-binding protein GsiB	ABC transporter complex (periplasmic space)	1.26	0.043
*K. oxytoca*	Phosphoglycerate kinase	ATP binding. Glycolytic process	0.37	0.030
*K. oxytoca*	Glycine betaine-binding periplasmic protein	Transporter activity	1.81	0.010
*K. oxytoca*	Protein TolB	Bacteriocin transport. Uptake of colicins (group A)	1.50	0.029
*K. oxytoca*	Leucine-specific-binding protein	L-alpha-amino acid transmembrane transport.	2.16	0.002
*K. oxytoca*	Pyruvate kinase I	Glycolysis. Magnesium, potassium and ATP-binding.	0.23	0.038
*K. oxytoca*	Glutamine-binding periplasmic protein	Amino acid transport.	1.49	0.040
*K. oxytoca*	6-phosphogluconolactonase	Pentose phosphate pathway.	0.5	0.022
*K. oxytoca*	Osmotically-inducible lipoprotein E	Possibly involved in maintaining the structural integrity of the cell envelope.	4.75	0.002
*K. oxytoca*	Periplasmic serine endoprotease DegP	Degrades transiently denatured and unfolded proteins accumulated in the periplasm in stress conditions	4.86	0.002
*K. oxytoca*	Uncharacterized protein	Unknown	10	0.004
*K. oxytoca*	Periplasmic trehalase	Cellular hyperosmotic response	3.60	0.049
*K. oxytoca*	Uncharacterized protein	Unknown	6.67	0.011
*K. oxytoca*	Uncharacterized protein	Unknown	0.13	0.031
*K. oxytoca*	Peptidoglycan-associated lipoprotein	Possibly bacterial envelope integrity	3.20	0.019
*K. oxytoca*	Chaperone protein MrkB	Cell wall organization. Chaperone-mediated protein folding. Pilus organization.	5.33	0.045
*K. oxytoca*	MltA-interacting protein	Peptidoglycan byosynthetic process	24	<0.001
*K. oxytoca*	Thiamine-binding periplasmic protein	ATP binding. Thiamine transport.	8	0.010
*B. thuringiensis*	Uncharacterized protein	Unknown	14.61	0.001
*B. thuringiensis*	Sulfatase	Sulfuric ester hydrolase activity	3.59	0.008
*B. thuringiensis*	Internalin	The function of this domain is not clear	4.12	<0.001
*B. thuringiensis*	Endopeptidase lytE, putative lytE	The function of this domain is not clear	2.08	0.023
*B. thuringiensis*	Peptidase M4 thermolysin	The function of this domain is not clear	2.70	<0.001
*B. thuringiensis*	Enolase	Glycolytic process	4.44	0.043
*B. thuringiensis*	Bacillolysin	Extracellular zinc metalloprotease	4.40	0.002
*B. thuringiensis*	Extracellular solute-binding protein family 5	The function of this domain is not clear	3.37	0.005
*B. thuringiensis*	VanW	Vancomycin resistan protein W. The function is not clear.	2.80	0.045
*B. thuringiensis*	Uncharacterized protein	Unknown	3.76	0.014
*B. thuringiensis*	Uncharacterized protein	Unknown	25	<0.001
*B. thuringiensis*	Uncharacterized protein	Unknown	5.33	0.026
*B. thuringiensis*	Uncharacterized protein	Unknown	14	0.011
*B. thuringiensis*	Oligopeptide transporter, periplasmic-binding protein	ABC transporter complex	17	0.001
*B. thuringiensis*	Periplasmic component of efflux system	Transmembrane transport	18	<0.001
*B. thuringiensis*	Camelysin Metallo peptidase MEROPS family M73	The function of this domain is not clear	7.33	0.031
*B. thuringiensis*	Flagellar hook-associated protein 3	Bacterial-type flagellum-dependent cell motility	11	0.014
*B. thuringiensis*	Uncharacterized protein	Unknown	10	0.035
*B. thuringiensis*	Uncharacterized protein	Unknown	8	0.010
*B. thuringiensis*	Uncharacterized protein	Unknown	7	0.021
*B. thuringiensis*	Foldase protein PrsA 2	Protein folding	9	0.008
*B. thuringiensis*	Glucose-specific phosphotransferase enzyme IIA component	Phosphoenolpyruvate-dependent sugar phosphotransferase system	0.19	0.030

## Discussion

Our present co-culturing approach represents an opportunity to study changes in protein diversity as a result of inter-bacterial relationships that can, to a certain extent, reflect the natural situation in a wound. In principle, the *in vitro* growth characteristics of *S. aureus* colonies over *B. thuringiensis* or *K. oxytoca* colonies suggest adaptive mechanisms for all species. From the proteome perspective, one mechanism that stands out is the depletion of staphylococcal cytoplasmic proteins from the exoproteomes of co-cultures upon co-culture with Bt or Ko. Proteomic analysis also revealed two uniquely detectable exoproteins in the co-cultures with Bt. The exoprotein found in the co-culture of *S. aureus* t111+Bt was SecG, a subunit of the heterotrimeric complex SecYEG. This complex serves as a membrane channel for translocation of secretory proteins in an unfolded state [31]. In *S. aureus*, SecG is of importance since *secG* deletion mutants displayed impaired secretion of several wall-bound proteins and abundant exoproteins [32]. Despite its known function in protein secretion, the reason for the unique detection of SecG in the t111-Bt exoproteomes is presently not clear. Additionally, in *S. aureus* t13595+Bt co-cultures an uncharacterized protein that resembles a control factor of the competence regulator ComK, named YlbF/YmcA, was found. This protein is known to be involved in biofilm formation by other Gram-positive and archaeal organisms. Further characterization of these two proteins might help us to understand why they are present in the exoproteome when *S. aureus* is co-cultured with Bt.

While the exoproteome of *S. aureus* in monoculture was previously found to contain numerous typically cytoplasmic proteins [17, 33–38], this is not an exclusive trait of *S. aureus*. The exoproteomes of other Gram-positive bacteria, such as *B. subtilis, Clostridium difficile,Corynebacterium diphtheria*, Group A *Streptococcus, Mycobacterium tuberculosis* and *Streptomyces lividans*, but also Gram-negative bacteria like *Porphyromonas gingivalis*, have been associated with high numbers of such typically cytoplasmic proteins.[36, 39–43] In *B. subtilis*, this observation has been explained by a combination of high cell lysis propensity, increased cell wall turnover and/or autolysin activity [44]. It has also been proposed that the ‘liberation’ of typical cytoplasmic proteins that lack the prototype signal peptides of proteins exported via the general Sec pathway for protein secretion could be facilitated by as yet unidentified alternative pathways, also referred to as ‘non-classical secretion systems’ [45–50]. For example, it is conceivable that, besides the well-characterized endolysins, the expression of prophage-encoded holins or holin-like proteins might have a role in the liberation of otherwise cytosolic proteins [42,50]. The release of cytosolic proteins due to the large pores formed by holins is still somewhat debated, since it has not been extensively studied in Gram-positive organisms. Nevertheless, the presence of additional holins could, thus, at least partially explain the elevated number of cytoplasmic proteins found in the growth medium of the *S. aureus* t13595 isolate compared to the t111 isolate. Yet, it has been shown for *B. subtilis* that engineered strains lacking all prophages still release typical cytoplasmic proteins into their growth medium [52]. Clearly, for the bacterial isolates investigated in the present study, it remains to be defined which particular mechanisms contribute, individually or synergistically, to the observed non-classical secretion phenomenon and to what extent.

Interestingly, the common denominator between the presently identified *S. aureus* exoproteome and that of its ‘wound mates’ is the presence of housekeeping enzymes, particularly glycolytic enzymes. For instance, glyceraldehyde-3-phosphate dehydrogenase (GAPDH) found in the *S. aureus* t13595, Bt and Ko cultures, has also been located on the surface of other pathogenic bacteria like group A streptococci, and enteropathogenic *E. coli* where it can serve either as a binding protein or as signal transduction initiator in epithelial cells [53–55]. Cell wall-associated proteins like enolase (displayed in the same cultures) have been reported to bind to plasminogen both in Gram-positive (*Streptococcus pneumoniae*) and Gram-negative (*Actinobacillus actinomycetemcomitans*) bacteria [56, 57]. Other multifunctional ‘moonlighting’ proteins like fructose-bisphosphate aldolase, identified in *S. aureus* t111 cultures, are recognized to play a role in adhesion to mammalian cells and binding of human plasminogen by several bacterial species, like *S. pneumoniae, M. turberculosis*, and *Neisseria meningitidis* [58]. Similarly, the elongation factor (EF) Tu, a guanosine nucleotide binding protein important for protein synthesis, was detected on the surface of *Mycoplasma pneumoniae* and *Lactobacillus johnsonii*, where it was involved in fibronectin- and mucin-binding, respectively [59, 60]. Furthermore, an immunoproteomic study of bacteremia showed that six housekeeping enzymes and EF-Tu were linked to leukopenia caused by *Klebsiella pneumonia* [61]. Altogether, these findings imply that proteins, which were originally considered to be restricted to the cytoplasm, may also be important for virulence enhancement and invasive disease [50, 62]. Indeed, for *S. aureus*, such proteins have previously been implicated in virulence, epidemiology and intracellular survival [46–48, 50, 63].

By tracking the *S. aureus*-specific exoproteome, we observed that especially the cytoplasmic protein fractions were depleted from the exoproteome in co-cultures. This suggests that there might be either an altered localization, decreased cell lysis, enhanced proteolysis or consumption of these proteins by the other organism in the respective co-culture. In the literature, a loss of the cytoplasmic exoproteome fraction was recently associated to the latter. When studying marine bacteria in mixed cultures, Christie-Oleza *et al.* showed that extracellular cytoplasmic proteins from *Synechococcus* are notably reduced when *Roseobacter* strains are present [64]. Their study also reported an abundance of active transporters and flagellar proteins of *Roseobacter* during co-culturing. Such a situation could potentially also apply to *K. oxytoca* and *B. thuringiensis* as the present data suggests that, when grown in a nutrient-limited environment, these bacteria might act as scavengers of carbon and nitrogen sources, particularly by degrading and assimilating extracellular staphylococcal proteins. If so, this would suggest that, at least in a closed system like the one we established *in vitro*, nothing goes to waste and that the carbon and nitrogen fixing characteristics of Bt and Ko are perhaps a distinguishable reflection of short-term interspecies interactions. Nevertheless, we cannot exclude the possibility that there could also be less *S. aureus* cell lysis during co-culture. One way to verify the idea that Bt and Ko consume extracellular proteins of *S. aureus*, may involve the metabolic labeling of *S. aureus* proteins with particular isotopes, providing these proteins to Bt or Ko cultures, and investigating the incorporation of the isotopes into Bt or Ko proteins or metabolites. Likewise, interactions between *S. aureus* and Bt or Ko could be investigated by transcriptomics and metabolomics. Irrespective of the molecular background of our observations, the depletion of *S. aureus* proteins from the exoproteome upon co-culturing has an important implication for the virulence of this bacterium as exoproteins should be considered as the prime reservoir of virulence factors [17, 50].

Beyond the known proteome heterogeneity [33, 34, 37]. and the genetic diversity of the colonizing staphylococcal strains [15, 65], we found a *mutL* mutation indicative of impaired mismatch repair and other hypermutable traits in our *S. aureus* t13595 isolate [66]. This finding allows us to place the relationships between the investigated *S. aureus* isolates in a broader perspective, where it is of note that the presently investigated bacterial isolates from an EB patient were obtained upon one-time sampling of a >8 months existing chronic wound. Importantly, it has been hypothesized that hypermutable strains play roles in species persistence and chronic infection [67, 68] and they could perhaps also favor the development of antibiotic resistance [69–71]. In the context of the long-term colonization of chronic wounds of EB patients by *S. aureus*, the influence of mutations on the intra-species heterogeneity should be considered in view of microbial social behavior and the consequences for fitness that particular mutations may provide [72]. Interestingly, with respect to the observed proteomic profiles, the *S. aureus* t111 isolate seems to foster a committed production of metabolically costly siderophores, virulence factors and cell adhesion molecules, while the t13595 isolate shows a more ‘recalcitrant’ performance focusing only on its metabolism and homeostasis. Moreover, during co-culturing of the t111 and t13595 isolates, we did not observe any protein overexpression, contrary to what was observed for the monocultures. This outcome is suggestive of a cooperative behavior. Therefore, it is tempting to hypothesize that the genetic variability as displayed by the ‘selfish’ t13595 isolate may be beneficial for the spread or persistence of *S. aureus* as a wound-colonizing species [73].

To the best of our knowledge, our research represents the first explanatory analysis of *S. aureus* exoproteomic shifts while in co-culture with wound-coexisting bacteria. Collectively, our findings point out the importance of studying bacteria in a broader biological context. It is evident that, for example, the growth rates of the four isolates explored here are not necessarily the same, and therefore it is not possible to compare the exoproteome of all investigated isolates at exactly the same growth stage. However, this situation is also true in a wound environment where not all co-existing microbes are completely synchronized or exist in equal numbers. Hence, the present data should be interpreted as one of many possible scenarios in which bacteria interact with each other with no apparent antagonistic relationships.

Because of the complexity of the wound environment and the peculiarities of each wound, it is difficult to establish a standard definition of the nutrients present in a wound. For example, temperature in a wound can influence local vasodilation and therefore the availability of nutrients. Furthermore, the biomolecules are primarily at the disposal of host cells rather than bacteria that have to compete for the nutrient restriction [74]. In the present investigations, we have presented a scenario that simulates the conditions in human plasma, which relies on bacteria cultivated under shaking and not static conditions. Accordingly, the present culture conditions may not exactly reflect the situation in chronic wounds of an EB patient. However, it seems reasonable to assume that in the wound environment, cytosolic bacterial proteins could represent an important foundation for microbiome cross talk and homeostasis. Altogether, our experiments introduce an attractive avenue to explore possible pre- and probiotic targets, such as quorum sensing- or quorum quenching-related factors or bacterial growth factors that can either hinder the invasion by pathogenic bacteria, or enhance the presence of non-pathogenic microbes that may prevent infections and could even enhance wound healing.

## Material and methods

### Ethical approval

The local medical ethics committee (METc) of the University Medical Center Groningen (UMCG) approved of the collection of non-invasive samples from patients with EB on the basis of obtained written informed patient consent. The experiments were conducted in accordance with the guidelines of the Declaration of Helsinki. All samples were anonymized.

### Bacterial isolation, identification and culture conditions

The used bandage from a chronic wound of a Dutch patient with Junctional Epidermolysis Bullosa was collected. The bandage was replica-plated with gentle pressure for 10 seconds onto CLED agar (Oxoid) using bioassay plates (245 × 245 × 245 ;mm; Nunc). After 48 h of incubation at 37°C, colonies were selected and replated onto blood agar plates for identification. Bacterial colonies were identified by matrix-assisted laser desorption/ionization-time-of-flight (MALDI-TOF) MS, using a MALDI Biotyper® (Bruker Corporation, Billerica, USA) as previously described [75]. All isolates were stored with 10% glycerol at −80°C.

To investigate whether the studied bacteria secrete compounds that interfere with each other's growth, blood agar plates were spread with pure overnight cultures of *S. aureus* isolates with an optical density at 600 nm (OD_600_) of 0.5 to obtain a confluent lawn of cells. Then, after 5 min, 10 µL aliquots of pure overnight cultures of *B. thuringiensis, K. oxytoca* or *Bacillus subtilis* 168 (OD_600_ 0.5) were applied to the center of the agar plate. For viable plate counting and the preparation of extracellular protein extracts, bacterial strains were grown overnight on Tryptic Soy Broth (TSB) under vigorous shaking at 37°C. The cultures were then diluted to an OD_600_ of 0.05 in pre-warmed RPMI medium without phenol red (GE Healthcare). Cultivation continued in a water bath under constant shaking (80–85 rpm) at 37°C until cultures reached an OD_600_ of ±0.5. Main cultures were again diluted in 120 mL pre-warmed RPMI medium under the same conditions. Monocultures were started with an initial OD_600_ of 0.05 while co-cultures were inoculated with an OD_600_ of 0.025 of each isolate to a total of 0.05. Given that an OD_600_ of 0.5 contained a total of ∼200 × 10^6^ colony forming units per mL (CFU/mL) for t111, t13595 and Ko, and ∼15 × 10^6^ CFU/mL for Bt, inoculated cultures corresponded to ∼20 × 10^6^ CFU/mL for t111, t13595 and Ko and to ∼1.5 × 10^6^ CFU/mL for Bt.

For proteome analyses and determination of CFU/mL, samples were collected at 90 min within the stationary growth phase. The ratios of different bacteria sampled from the co-cultures remained nearly the same with the exception of the co-cultures of *S. aureus and K. oxytoca*, where *K. oxytoca* grew 1.5 times faster than t111 and 1.7 times faster than t13595.

### Genomic DNA extraction, genome sequencing and analysis

Libraries for whole genome sequencing were prepared from *S. aureus* t111, t13595, *B. thuringiensis*, and *K. oxytoca* DNA with the Nextera XT v2 kit, and run on Illumina next generation sequencing platforms (MiSeq) according to the manufacturers' instructions (Illumina, San Diego, CA, USA). Read data for the study isolates have been deposited in the National Center for Biotechnology Information (NCBI) under accession number SRP094393. The respective .fastq files were submitted for *de novo* sequence assembly using CLC Genomics Workbench v7.0.4 (CLC bio A/S, Aarhus, Denmark) after quality trimming (Qs > 28) with optimal word sizes based on the maximum N50 value. *De novo* assembled contigs of the *S. aureus* isolates were imported into the Seqsphere+ software version 1.0 (Ridom GmbH, Würzburg, Germany) for *in silico* multi-locus sequence typing (MLST) and *spa* typing as previously described [76, 77]. Automatic genome annotation was done on the rapid annotation using subsystem technology (RAST) server 2.0 for all the genomes [78, 79]. *De novo* assembled genomes of sequenced isolates were queried against the closest related reference genome of *S. aureus* strain N315 (GenBank: NC_007795.1), *B. thuringiensis* serovar konkukian str. 97–27 (GenBank: NC_017200), and *K. oxytoca* strain E718 (GenBank: NC_018106) using blastN in the WebACT comparison tool (http://www.webact.org/WebACT/prebuilt#) and analyzed in detail by the Artemis Comparison Tool (ACT) software [80, 81]. Similarity matches were filtered based on their length (100 kb segments) and percentage similarity scores, and only the filtered hits with at least 80% sequence similarity were then displayed by ACT (e-value of 10.00000) and analyzed in detail.

### Preparation of protein extracts

All samples were collected in duplicate according to the growth curves of the *S. aureus* isolates after 90 min of stationary growth. Cells were removed by centrifugation (10 min, 8000 x *g*, 4°C) from 2 mL culture aliquots and the supernatant was subsequently filtrated with a 0.22 µm filter (GE Healthcare Systems, Little Chalfont, United Kingdom). Proteins were precipitated with 10% w/v TCA on ice at 4°C overnight. The precipitates were harvested by centrifugation (20 min, 8000 × *g*, 4°C) and washed with ice-cold acetone. The protein pellets were dried at room temperature and stored at −20°C until further use. Total protein concentration was determined with the Pierce BCA protein quantification assay (ThermoFisher Scientific). Protein pellets were suspended in 50 mM ammonium bicarbonate buffer (Fluka, Buchs, Switzerland). As reducing agent, 10 mM dithiothreitol (Duchefa Biochemie, Haarlem, the Netherlands) was used for 30 min, and as alkylating agent, 10 mM iodoacetamide (Sigma-Aldrich, St. Louis, USA) was used for 30 min in the dark. Further, overnight incubation with 80 ng of trypsin (Promega, Madison, USA) at 37°C was followed by the addition of 0.1% trifluoroacetic acid (TFA, Sigma-Aldrich, St. Louis, USA) to end the digestive reaction. The samples were desalted by Zip-Tip purification (Millipore, Billerica, USA) as previously described by Dreisbach et al. [82]. The final eluates were concentrated using a vacuum centrifuge (Eppendorf, Hamburg, Germany) and stored at 4°C until further use.

### Mass spectrometry and data analysis

Tryptic peptides were separated by reversed phase liquid chromatography (LC). LC-MS/MS analyses were performed using a nanoACQUITY UPLC system (Waters, Milford, MA) coupled to an LTQ XL Orbitrap mass spectrometer (Thermo Fisher Scientific,Waltham, MA) creating an electro spray ionization through a Picotip Emitter (SilicaTip, FS360-20-10 Coating P200P, New Objective). Specifically, the peptides were loaded onto a trap column (Symmetry C18, 5 μm, 180 μm inner diameter × 20 mm, Waters). Elution was performed onto an analytical column (BEH130 C18, 1.7 μm, 100 μm inner diameter × 100 mm, Waters) by a binary gradient of buffer A (0,1% (v/v) acetic acid) and B (0,1% (v/v) acetic acid in acetonitrile) over a period of 80 min with a flow rate of 400 nL min^−1^. A stepped gradient was applied.

For MS/MS analysis a full survey scan was recorded in the Orbitrap (m/z 300–2000) with a resolution of 30,000. The five most abundant precursor ions from each survey scan were consecutively isolated in the LTQ XL and fragmented via collision induced dissociation (CID). Precursors were dynamically excluded for 30 s and unassigned charge states as well as singly charged ions were rejected [83]. Internal calibration was applied (lock mass 445.120025 m/z). Database searching was done with Sorcerer-SEQUEST 4 (Sage-N Research, Milpitas, USA). After extraction from the raw files, *.dta files were searched with Sequest against a concatenated target-pseudoreversed decoy database with a set of common laboratory contaminants. The target database was the Uniprot reference database (downloaded on September 2014) of *B. thuringiensis* (5815 protein hits)*, K. oxytoca* (5663 protein hits), *S. aureus* t111 (2574 protein hits) and *S. aureus* t13595 (2625 protein hits). The *.out files were compiled with Scaffold 4 (version Scaffold_4.4.1, Proteome Software Inc., Portland, OR). Protein identification criteria were carried out as specified by Hempel *et al.*, [83]. but with the following XCorr values: ≥ 2.2, 3.3, 3.75 for doubly, triply and higher charged peptides. To allow protein abundance comparisons between samples, Scaffold MS/MS data were normalized by adjusting the sum of the selected quantitative values for all proteins in the list within each MS sample to a common value: the average of the sums of all MS samples present in the experiment. This was achieved by applying a scaling factor for each sample to each protein or protein group adjusting in this way the selected value to a normalized “Quantitative Value” [84]. These values were exported from Scaffold and further curated in spreadsheets for analysis (Table S3). The MS raw data has been deposited to the ProteomeXchange Consortium via the PRIDE partner repository with the dataset identifier PXD005596 [Username: reviewer87282@ebi.ac.uk / Password: byH5iQGT]. Volcano plots used the normalized values for calculating the fold-changes in each protein among the monoculture samples versus the co-culture samples.

### Bioinformatic analyses

Subcellular protein localization was predicted with TMHMM (version 2.0) [85, 86], SignalP (version 4.1) [87], LipoP (version 4.1) [88], PSORTb (version 3.0) [89], and ClusbSub-P (version 2.18.3) [90]. Proteins with ambiguous predictions were manually curated by inspection of their individual sequences with InterProScan 5 (version 49.0) [91]. For interpretation and visualization of biologically relevant protein functions, the gene ontology (GO) terms identified for the investigated exoproteomes were imported into the freely available online Revigo software (http://revigo.irb.hr/) [92].

### Biological and chemical safety

*S. aureus* and *K. oxytoca* are biosafety level 2 (BSL-2) microbiological agents and were accordingly handled following appropriate safety procedures. All experiments involving live bacteria and chemical manipulations of bacterial protein extracts were performed under appropriate containment conditions. All chemicals and reagents used in this study were handled according to the local guidelines for safe usage and protection of the environment.

## Supplementary Material

New_folder__3_.zip
