# Supplementary material for: From the wound to the bench: exoproteome interplay between wound-colonizing Staphylococcus aureus strains and co-existing bacteria
Source: Virulence. 2018 Mar 1;9(1):363–78. doi: 10.1080/21505594.2017.1395129 (PMC5955179; doi:10.1080/21505594.2017.1395129)
Supplement: New_folder__3_.zip [file kvir-09-01-1395129-s001.zip › New folder (3)/2017VIRULENCE0206R1-f05-z-4c.pdf]

From the wound to the bench: exoproteome interplay between the *Staphylococcus aureus* strains and co-existing bacteria

Andrea N. García-Pérez[1], Anne de Jong[2], Sabryna Junker[3], Dörte Becher[3], Monika Chlebowicz[1], José Duipmans[4], Marcel F. Jonkman[4], and Jan Maarten van Dijl[1]*

*Corresponding author, E-mail: j.m.van.dijl01@umcg.nl, Phone: +31-50-3615187

[1]Department of Medical Microbiology, University of Groningen, University Medical Center Groningen, Groningen, The Netherlands
[2]Department of Molecular Genetics, Groningen Biomolecular Sciences and Biotechnology Institute, University of Groningen, Groningen, The Netherlands
[3]Institute for Microbiology, Ernst-Moritz-Arndt-University of Greifswald , Greifswald, Germany
[4]Department of Dermatology, University Medical Center Groningen, University of Groningen, Groningen, The Netherlands

- Figure S1. Growth curves in RPMI
- Figure S2. Reproducibility of the MS/MS results
- Figure S3. Protein gel and absolute protein quantification
- Figure S4. Exoproteome subcellular localization
- Figure S5. Venn diagrams of predicted protein functions found in the mono and co-cultures of *K. oxytoca* and *B. thuringiensis*
- Figure S6. Volcano plots of the normalized spectral counts
- Table S1. *S. aureus* t111 and t13595 resistome.
- Table S2. Summary of mobile genetic elements (MGE) in sequenced *S. aureus* t111 and t13595 strains associated with virulence or drug resistance
- Table S3. Master Table with exoproteome report displaying quantitative values (normalized total spectra)
- Table S4. Relationship among cultures. The table describes the proteins present only in monoculture or only in co-culture and proteins common in two or more cultures.

Figure S1

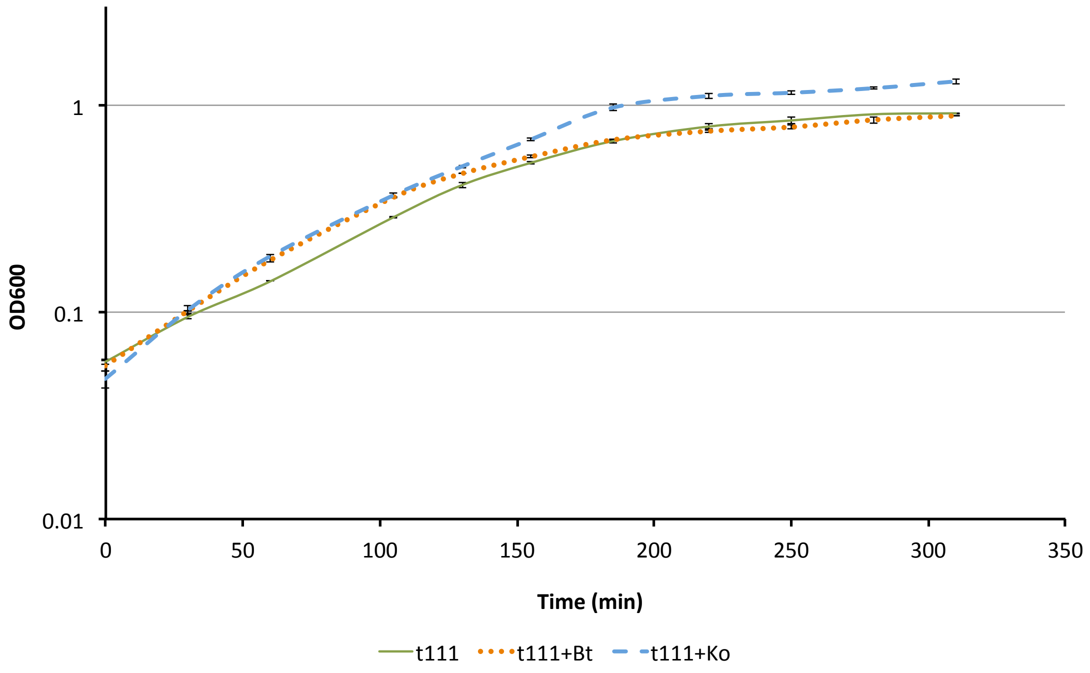

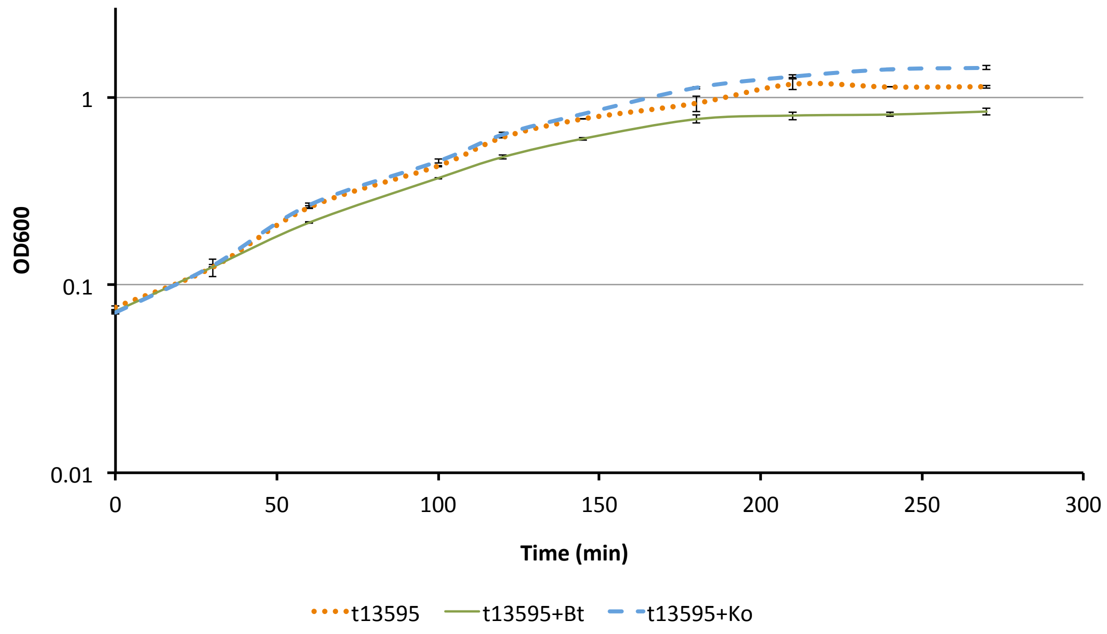

Figure S1. **Growth curves of *S. aureus* monocultures and co-cultures with *B. thuringiensis* (Bt) or *K. oxytoca* (Ko) in RPMI medium under vigorous shaking at 37ºC.** (A) growth curves of *S. aureus* t111. (B) growth curves of *S. aureus* t13595.

Figure S2

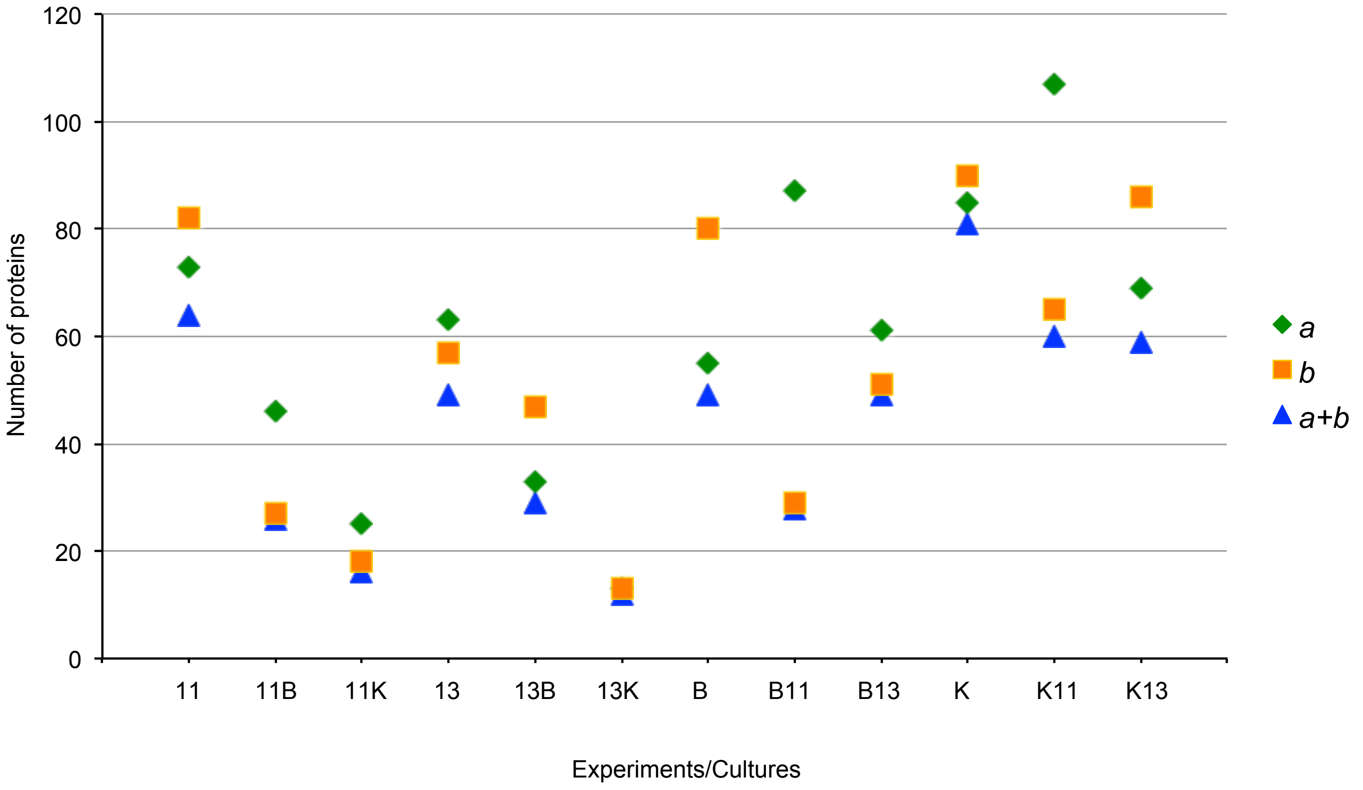

Figure S2. Reproducibility of the MS/MS results. Letters ***a*** and ***b*** represent the number of proteins found separately in each biological replicate of the same experiment. Proteins present in both replicates are represented as ***a+b***. In this case, when a protein was found only in one biological replicate, it was not considered. The legend on the horizontal axis refers to the proteins of *S. aureus* t111 in monoculture (11) and in co-culture with *B. thuringiensis* (11B) and *K. oxytoca* (11K). The same applies for *S. aureus* t13595 (13, 13B, 13K), *B. thuringiensis* (B, B11, B13), and *K. oxytoca* (K, K11, K13) cultures.

Figure S3

A

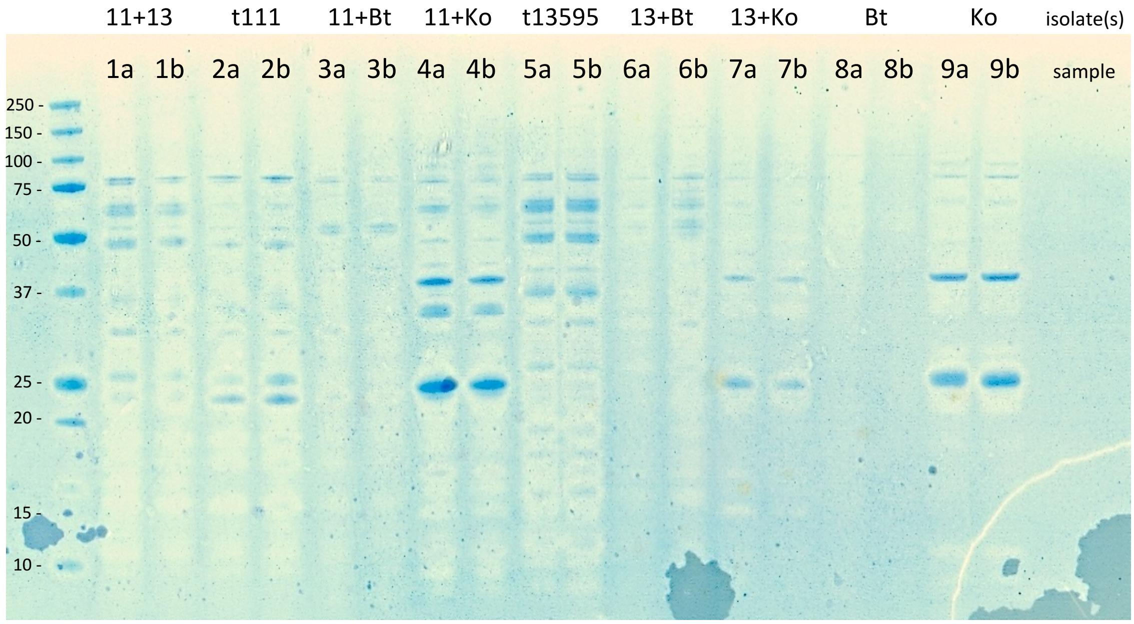

B

Monocultures

culture	t111 (11)	t13595 (13)	Bt	Ko
ug/mL	62	46	20	34

Co-cultures

culture	11+13	11+Ko	11+Bt	13+Ko	13+Bt
ug/mL	69	27	28	30	27

Figure S3. Protein amounts in mono- and co-cultures. (A) SDS-PAGE gel stained with Coomassie blue dye. Exoprotein samples were collected at 90 min into the stationary growth phase. All mono and co-culture samples are represented in the gel: 1a & 1b= *S. aureus* t111 + *S. aureus* t13595; 2a & 2b= *S. aureus* t111; 3a & 3b= *S. aureus* t111 + *B. thuringiensis*; 4a & 4b= *S. aureus* t111 + *K. oxytoca*; 5a & 5b= *S. aureus* t13595; 6a & 6b= *S. aureus* t13595 + *B. thuringiensis*; 7a & 7b= *S. aureus* t13595 + *K. oxytoca*; 8a & 8b= *B. thuringiensis* and 9a & 9b= *K. oxytoca*. **(B)** Protein concentration as determined with the BCA assay method.

A

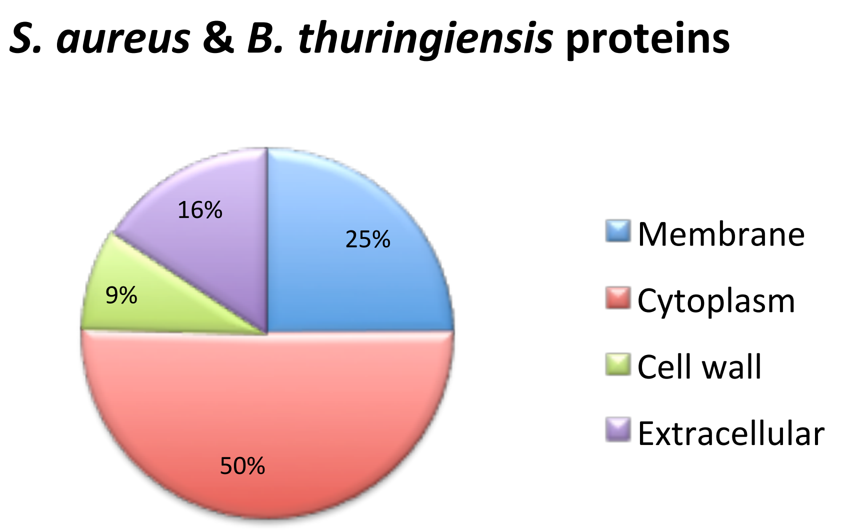

B

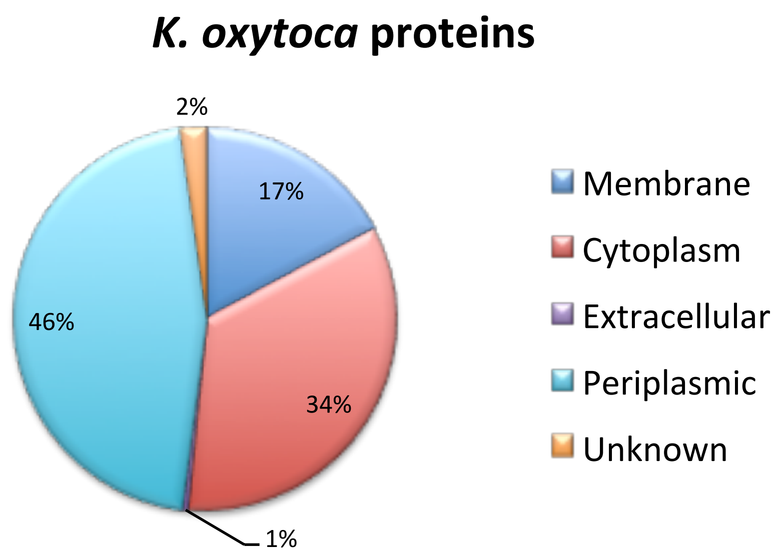

Figure S4: Exoproteome subcellular localization. Prediction of the extracellular localization of **(A)** Gram-positive and **(B)** Gram-negative species.

Figure S5

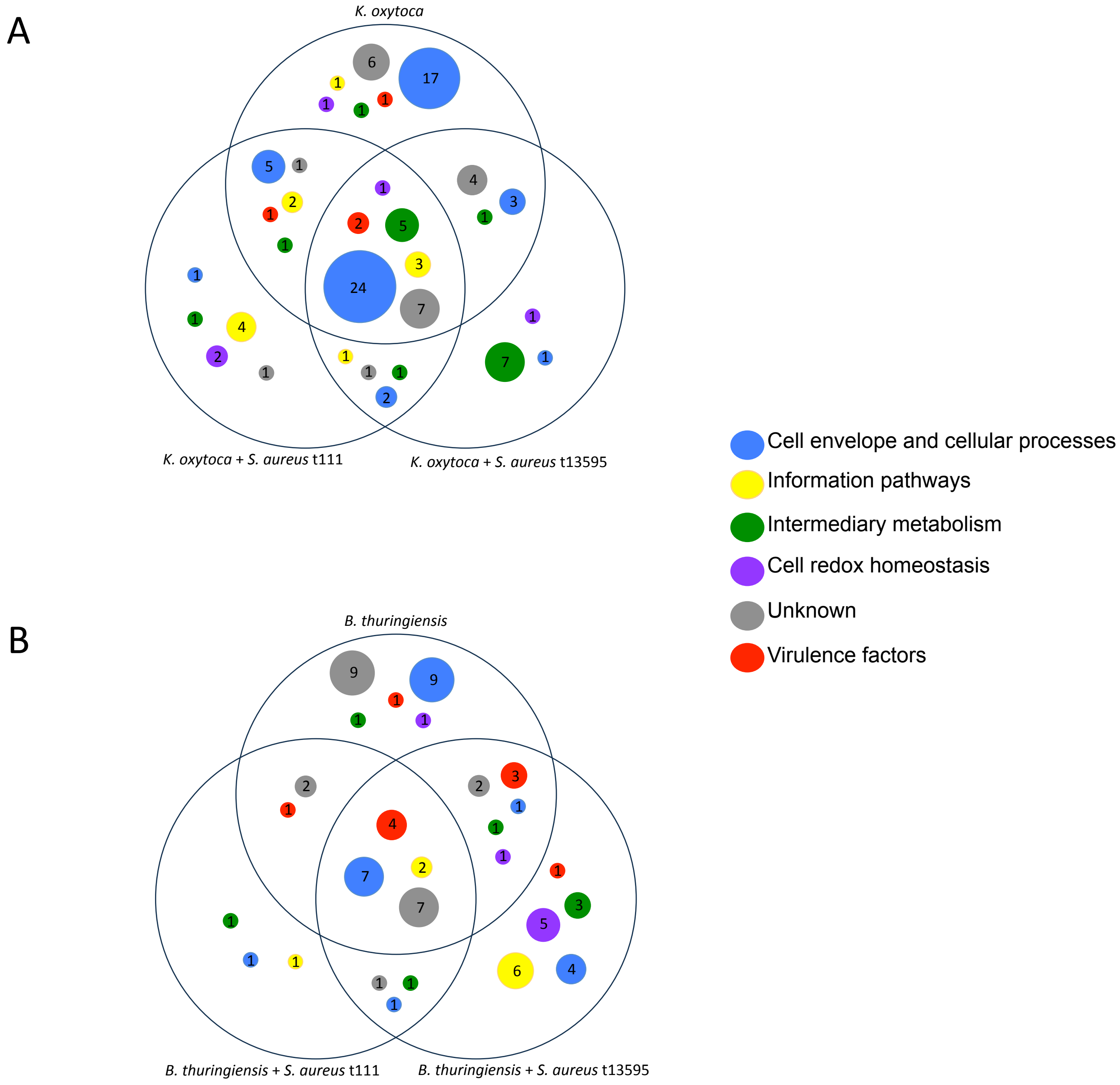

Figure S5: Venn diagrams of predicted protein functions found in *K. oxytoca* and *B. thuringiensis*. Diagram (A) depicts only *K. oxytoca* proteins. The area on the top shows the proteins detected only in monoculture, while the lower left area shows 9 proteins detected when *K. oxytoca* was co-cultured with t111. Likewise, diagram (B) shows exclusively the proteins that belong *B. thuringiensis* in monoculture and co-culture with t111 and t13595.

Figure S6

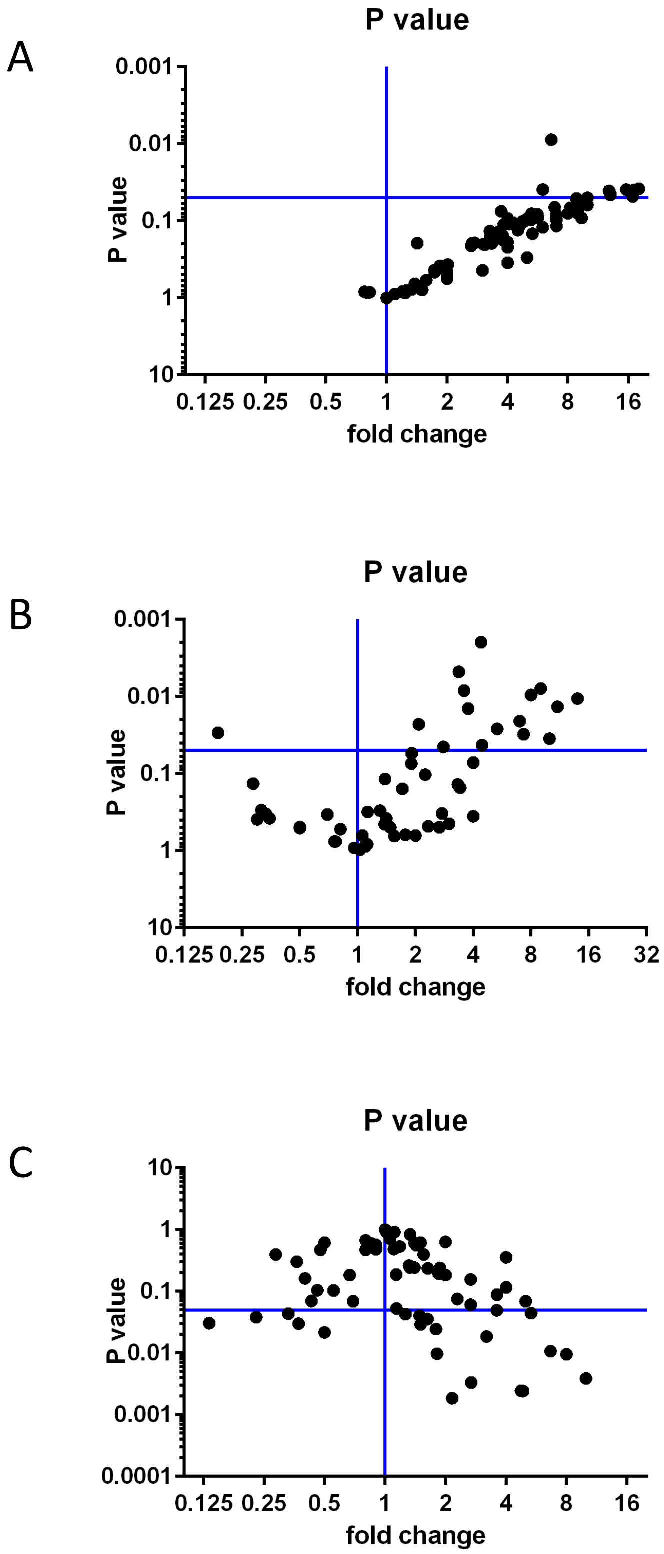

Figure S6: Volcano plots of the normalized spectral counts. For each mono- and co-culture, a quantitative estimate was calculated using volcano plots. The plots display P values (-$\log_{10}$) versus fold changes ($\log_2$) of the normalized spectral counts. These values projected ‘upregulated proteins’ either in monoculture (fold changes >1) or in co-cultures (fold changes ≤1). A) *Staphylococcus aureus* cultures. B) *Bacillus thuringiensis* cultures. C) *Klebsiella oxytoca* cultures.
